# A genome-wide portrait of pervasive drug contaminants

**DOI:** 10.1038/s41598-021-91792-1

**Published:** 2021-06-14

**Authors:** Joseph Uche Ogbede, Guri Giaever, Corey Nislow

**Affiliations:** 1 Genome Science & Technology Graduate Program, University of British Columbia, Vancouver, Canada; 2 Faculty of Pharmaceutical Science, University of British Columbia, Vancouver, Canada

**Keywords:** Functional genomics, Genomics, Pharmacology, Toxicology, Chemical genetics

## Abstract

Using a validated yeast chemogenomic platform, we characterized the genome-wide effects of several pharmaceutical contaminants, including three N-nitrosamines (NDMA, NDEA and NMBA), two related compounds (DMF and 4NQO) and several of their metabolites. A collection of 4800 non-essential homozygous diploid yeast deletion strains were screened in parallel and the strain abundance was quantified by barcode sequencing. These data were used to rank deletion strains representing genes required for resistance to the compounds to delineate affected cellular pathways and to visualize the global cellular effects of these toxins in an easy-to-use searchable database. Our analysis of the N-nitrosamine screens uncovered genes (via their corresponding homozygous deletion mutants) involved in several evolutionarily conserved pathways, including: arginine biosynthesis, mitochondrial genome integrity, vacuolar protein sorting and DNA damage repair. To investigate why NDMA, NDEA and DMF caused fitness defects in strains lacking genes of the arginine pathway, we tested several N-nitrosamine metabolites (methylamine, ethylamine and formamide), and found they also affected arginine pathway mutants. Notably, each of these metabolites has the potential to produce ammonium ions during their biotransformation. We directly tested the role of ammonium ions in N-nitrosamine toxicity by treatment with ammonium sulfate and we found that ammonium sulfate also caused a growth defect in arginine pathway deletion strains. Formaldehyde, a metabolite produced from NDMA, methylamine and formamide, and which is known to cross-link free amines, perturbed deletion strains involved in chromatin remodeling and DNA repair pathways. Finally, co-administration of N-nitrosamines with ascorbic or ferulic acid did not relieve N-nitrosamine toxicity. In conclusion, we used parallel deletion mutant analysis to characterize the genes and pathways affected by exposure to N-nitrosamines and related compounds, and provide the data in an accessible, queryable database.

## Introduction

Although our bodies are exposed to thousands of diverse chemical toxins, our understanding of their mechanisms of action is limited. Further complicating the characterization of the threat posed by such contaminants is the fact that exposure levels can vary widely, and—as Paracelsus reportedly said, the “the dose makes the poison”. Exactly how chemical contaminants exert their toxic biological effects is often complex, involving multiple cellular reactions which impinge on many targets and pathways rather than a single cellular target^1^.

One source of human exposure to chemical toxins is via pharmaceutical contaminants introduced during the manufacturing of drugs—and recent cases of widespread drug contamination by N-nitrosamine provide a cautionary tale. In 2018, international drug regulators (including the European Medicines Agency, Health Canada and the US Federal Drug Administration) ordered a recall of sartan-type drugs after they detected contamination with N-nitrosodimethylamine (NDMA), a genotoxic and carcinogenic toxin. N-nitrosodiethylamine (NDEA) and N-methyl-N-aminobutyric acid (NMBA) were subsequently identified in lots of sartan drugs from several manufacturers. These contaminants were initially detected in the active pharmaceutical ingredients (APIs), and subsequently also attributed to the solvents used in their production and drug breakdown during storage^2,3^. Since the initial sartan recalls, other drug classes including metformin and ranitidine have also been recalled due to unacceptably high levels of NDMA. These probable carcinogens^4^ have been studied in cooked foods, treated water, tobacco products, industrial by-products and at sites of environmental pollution^5,6^. N-nitrosamines can also be formed endogenously during the course of metabolic transformation^7,8^. As a class, these contaminants have been implicated in diverse cancers in at least 40 different experimental animals^9^.

While the level of N-nitrosamine contamination found in some of these medicines has been reported to be relatively low, little is known regarding the long-term consequences of low-level N-nitrosamine ingestion. An 8-week study of ~ 5000 Danish patients failed to find evidence for a marked increase in short-term overall risk of cancer in patients taking NDMA-contaminated valsartan, yet the authors cautioned that there is uncertainty about single cancer outcomes, and that studies with longer follow-up are needed to assess long-term cancer risk^10^.

Despite decades of study on the toxicity of N-nitrosamines and N-nitrosamino acids, our understanding of how these toxins exert their biological effects on cellular systems is limited, hindering our ability to develop a comprehensive view of any potential harms and impeding the development of countermeasures. To address the dearth of data regarding which genes and pathways are perturbed by N-nitrosamine toxicity, we applied an unbiased chemogenomic assay that quantifies the requirement for each gene for resistance to a compound in vivo^11–13^. Our results uncovered multiple pathways that could be affected by N-nitrosamines and related compounds in yeast, including genes involved in arginine biosynthesis, mitochondrial function and DNA damage repair. The results are presented in a simple-to-use web application for visualizing these genes and pathways.

## Results

### N-nitrosamine-gene interactions visualized using a chemogenomic screen

We used the yeast chemogenomic assay^12,14^ to systematically screen ten compounds: NDMA, NDEA, NMBA, N,N-dimethylformamide (DMF), 4-nitroquinoline-1-oxide (4NQO), methylamine, ethylamine, formamide, formaldehyde and ammonium sulfate. In this assay, a pool containing equal amounts of each of 4800 diploid yeast strains carrying a precise start-to-stop homozygous deletion of a single gene^15^, are challenged with a compound at a low inhibitory dose (~ 20% inhibition relative to the wild type) and grown competitively. A key feature of each homozygous diploid deletion strain is that they are tagged with two unique 20 bp sequences (‘uptag’ and ‘downtag’) that serve as strain identifiers (‘barcodes’), allowing relative strain abundance to be quantitatively assessed by sequencing the ‘barcodes’^16^. The result is a list of each strain’s fitness (versus either dimethyl sulfoxide or water controls) allowing each deleted gene to be ranked in order of its importance for growth and survival. This quantitative metric describes the ‘fitness defect’ (FD) score, which is expressed as the − log_2_ ratio of each individual strain’s abundance relative to control. In the experiments presented here, we set the FD score significance threshold at 1.0, to capture any strain that is at least two-fold more sensitive to treatment versus the control. Analysis of these ranked gene lists allowed us to (1) identify those involved in buffering the cell’s exposure to each compound toxicity, (2) compare each genome-wide signature using hierarchical clustering, and (3) use gene set enrichment analysis to visualize these results.

**Figure 1:**
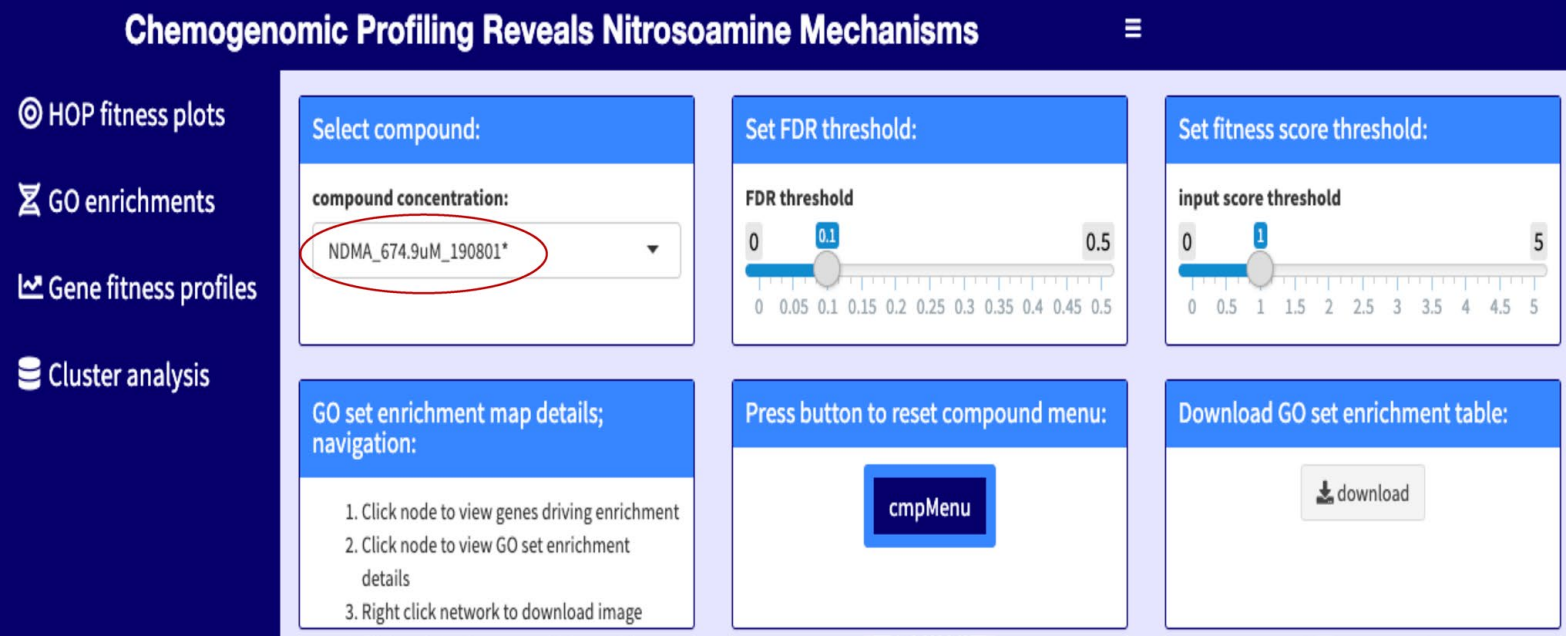
Layout of the interactive chemogenomic web application. Selecting a compound of interest from the drop-down list (red circle), will allow one to explore (left panel) its fitness profile, GO enrichment profile, individual gene fitness profile (cofitness) or cluster analysis of all the compounds in our screens. Compounds were saved with their final concentration and the date (yy/mm/dd) when the count matrices were performed e.g. NDMA_674.9µM_190801. To allow for custom visualizations, the user can adjust thresholds for FDR and fitness scores. Image is a screenshot from the web application.

We developed an interactive chemogenomic web application (https://ggshiny.shinyapps.io/2020NitrosoMechanisms/) that allows one to perform several visualizations, including Gene Ontology (GO) enrichment, fitness defect score ranking, scatterplot generation, and cofitness and coinhibition rankings. We used this application to visualize the results from our genome-wide experiments and to define sensitive genes and genetic pathways that were either shared between experiments or unique to a particular treatment. This application allows one to compare the data from our fitness screens to orthologous datasets (such as co-expression or proteomic surveys). The application dashboard is shown in Fig. 1 with key features highlighted, and an introductory video is provided to guide users (Supplementary Movie S1).

### Growth of several deletion strains is perturbed by treatment with N-nitrosamines

**Figure 2:**
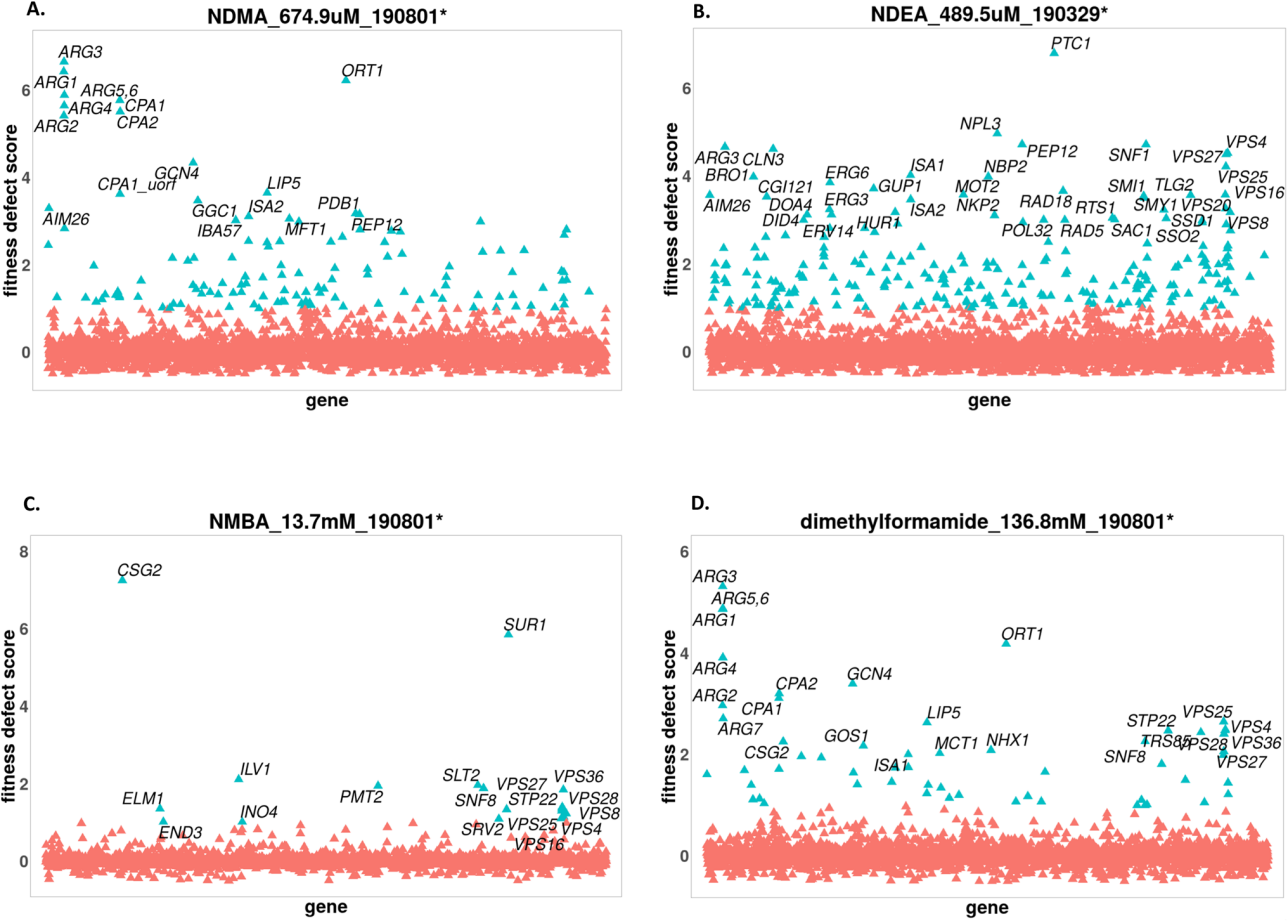
Fitness profiles showing genes (via deletion strains) affected by nitrosamines and related compounds. Genes (arranged alphabetically) are plotted on the horizontal axis versus their corresponding fitness defect (FD) scores on the vertical axis. Sensitive strains have a positive FD score, while resistant strains have a negative FD score. (**A**) Sensitive strains affected by NDMA include those lacking *ARG1*, *ARG3*, *GCN4*, *LIP5*, *AIM26* etc. (**B**) Genes affected by NDEA (via effect on their deletion strains) are *PEP12*, *ARG3*, *SNF1*, *BRO1*, among others. (**C**) *CSG2*, *SUR1*, *ILV1*, *PMT2* etc. have their deletion strains affected by NMBA. (**D**) DMF was sensitive to strains lacking the following genes *ARG3*, *GCN4*, *ORT1*, *SNF8* etc. The concentration at which the compound was screened is indicated. Not all strains (genes) with significant FD scores are labelled. Fitness profiles were generated using the Bioconductor v3.12, https://www.bioconductor.org/.

**Figure 3:**
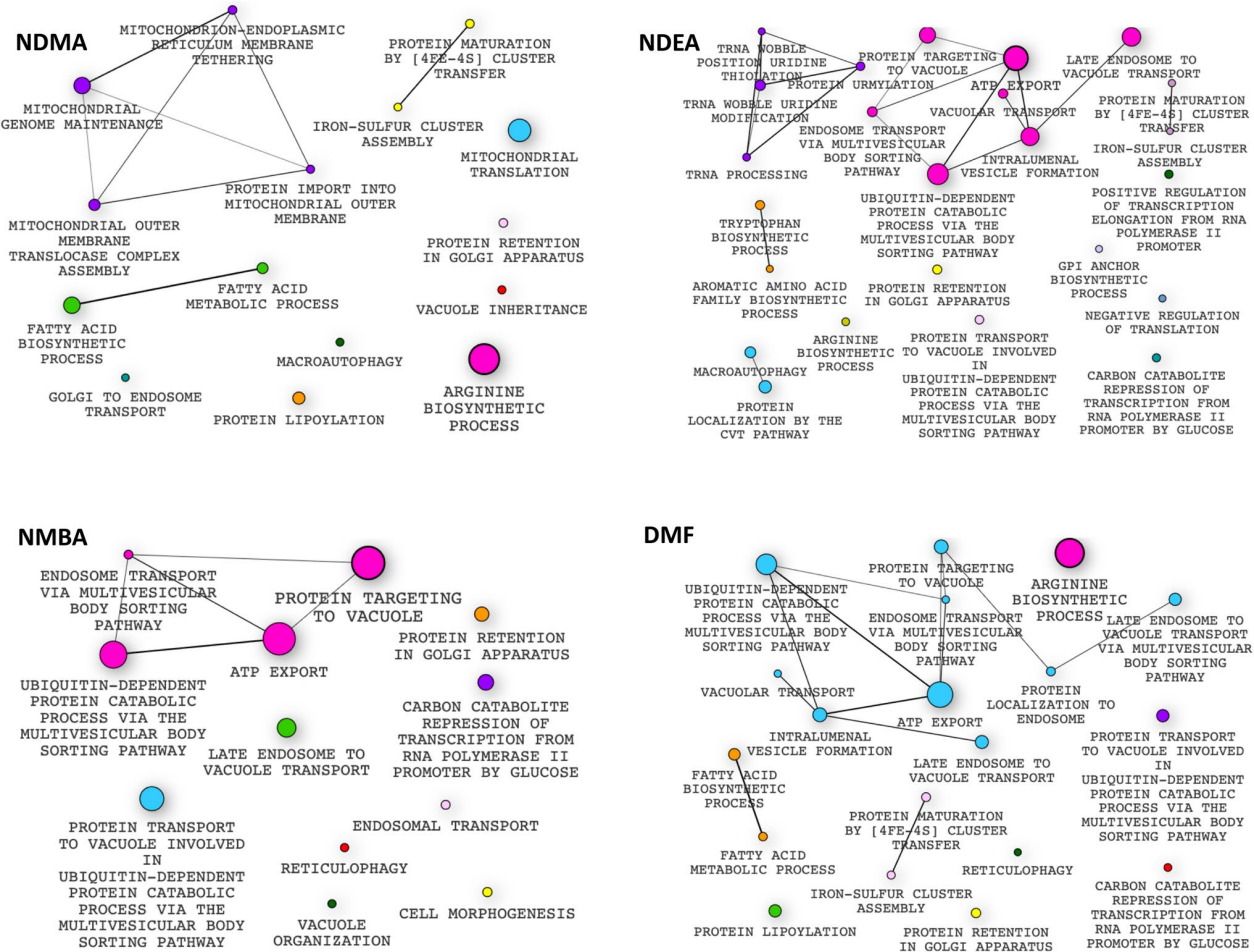
Gene Ontology enrichment profiles for the nitrosamines and DMF. Genes lacking in the sensitive strains were enriched for certain biological processes including; NDMA: arginine biosynthesis, fatty acid metabolic process, mitochondrial genome maintenance and macroautophagy. NDEA: protein transport, vacuolar transport, arginine biosynthesis, negative regulation of translation and macroautophagy. NMBA: protein transport, protein targeting to vacuole and cell morphogenesis. DMF: arginine biosynthesis, protein transport and iron–sulfur cluster assembly. Each node represents a gene set while the node size represents the number of genes in the gene set. The edge represents overlap between gene sets while the edge width represents the number of genes that overlap between connected gene sets. Not all pathways enriched at significant FD score are shown, e.g. septin ring assembly for NDEA, etc. Enrichment profiles were generated using the Bioconductor v3.12, https://www.bioconductor.org/.

Our results showed the following tally of deletion strains whose growth was sensitive to each of the three N-nitrosamines; NDMA—132, NDEA—254 and NMBA—22. The genes deleted in these sensitive deletion strains are involved in distinct biological pathways (Table 1; Figs. 2, 3). Notable mutant strains affected include those whose deleted genes are involved in vacuolar protein sorting (e.g. *VPS8*, *VPS16*, *VPS27* and *VPS36*). NMBA solely sensitized *ELM1* and *SRV2* mutants that lack genes required for cell morphogenesis. While NDMA and NDEA caused growth defect (fitness defect) of *RAD5* and *RAD18* deletions, NDEA uniquely decreased the growth of *RAD55* and *RAD57* deletion mutants, which are defective in DNA repair. Additionally, upon exposure to both NDMA and NDEA, we identified mutants whose deleted genes are required for mitochondrial genome maintenance, such as *HMI1*, *MDJ1*, *GGC1* and *MMM1*. Additional evidence for the requirement of an intact mitochondria is seen in our finding that strains lacking genes of the iron–sulfur cluster (*IBA57*, *ISA1* and *ISA2*) were hypersensitive to NDMA. Several strains deleted for genes that are components of the fatty acid biosynthesis and protein lipoylation pathways showed decreased growth in the presence of NDMA. For instance, in the case of fatty acid biosynthesis, the sensitive strains include those deleted for *OAR1*, *MCT1*, *PPT2*, *HTD2*, *CEM1*, *ETR1* and *HFA1*, while for protein lipoylation, strains with reduced growth include *AIM22*, *GCV3*, *LIP2* and *LIP5*. Strains lacking genes of the protein urmylation pathway showed fitness defect in NDEA, including *ELP2*, *ELP6*, *UBA4*, *URM1* and *NCS6*. The GO enrichment profiles showing the pathways affected by these compounds is shown in Fig. 3.

**Table 1:** An overview of major genes and their pathways (by nature of their deletion strains) affected by each of the 10 compounds screened.

Pathway	NDMA	NDEA	NMBA	DMF	Methylamine	Ethylamine	Formamide	NH_4_SO_4_	Formaldehyde	4NQO
ARGININE biosynthesis	ARG3, ARG1, ORT1, ARG5,6 CPA1, ARG4, CPA2, ARG2, ARG7	ARG3, ARG5,6, ARG1, ORT1, CPA1, CPA2		ARG3, ARG5,6, ARG1, ORT1, ARG4, CPA2, CPA1, ARG2, ARG7	ARG3, ARG1, ARG4, ARG5,6, ORT1, ARG2	ARG3	ARG3, CPA2, CPA1, ARG1, ORT1	ARG3		
Other amino acid metabolism	GCN4, PRO1	TRP3, TRP4, TRP1, TRP5, GCN4, HOM6, CYS4, CYS3	ILV1	GCN4	GCN4	THR1, HOM2	GCN4, ORT1, ARO2, CYS4	THR1, HOM3, HOM2, HOM6, THR4		THR1
								SER2, SER1, ALT1		
Chromatin remodeling	NPL6	NPL6, SNF5			YAF9, SNF6, SNF5, SNF2, SWC5, HTL1, SWR1, VPS71	SNF6, RSC2, SNF5	HTZ1, HTL1, YAF9, ARP6, SWC3, SWR1, VPS7, SWC5	SNF5		SNF2, ARP8, NPL6, ARP5, IES6, SNF5,, ARP6,
DNA repair and cell cycle or cell morphogenesis	RAD5, RAD18	RAD5, RAD18, RAD55, RAD57	ELM1, SRV2						SRS2, RAD18, RAD51, RAD55, RAD27, RRD1, MUS81	RAD2, RAD14, RAD7, RAD5, REV1
									CDC26, SWM1	DBF2, RAD9, PAT1
ATP export protein targeting to vacuole	VPS8, VPS45, VPS16, VPS9, VPS27, VPS36	VPS27, VPS28, VPS20, DID4, VPS2, SNF8, VPS36, SNF7, VPS24, STP22	SNF8, VPS36, VPS28, VPS27, STP22, VPS25, VPS8, VPS63, VPS16	VPS25, VPS36, STP22, VPS28, SNF8, VPS27, VPS20, DID4, BRO1, SNF7	VPS16, VPS41, CCZ1, MON1, VPS33, VPS71, VID24, STP22, APM3		VPS20, VPS28, SNF7, VPS36, SNF8, STP22, VPS25, DOA4	VPS45, VPS16, VPS1, APL6, APM3, VPS41, APS3	VPS8, VPS16	VPS9, VPS8, VPS45, SNF8, VPS64, STP22, VPS28, SNF7, VPS25
Fatty acid biosynthetic process	OAR1, MCT1, PPT2, HTD2, ETR1, HFA1, CEM1			MCT1, ELO3, PPT2, HTD2, CEM1						
Protein lipoylation or protein urmylation	LIP5, LIP2, AIM22, GCV3	ELP6, ELP2, UBA4, URM1, NCS6, NCS2		LIP5, GCV3, AIM22, LIP2						
Iron–sulphur cluster	ISA1, ISA2, IBA57	ISA1, ISA2, IBA57		ISA1, ISA2, IBA57						
Mitochondrial genome maintenance & translation	GGC1, MDJ1, MDM10, HMI1, GEP5, MEF2, RSM27, IMG1, RSM19, NAM2	MDJ1, MDM12, MDM34, HMI1, GGC1, MEF2, MRPL32, MRP7								

The comprehensive data can be individually assessed through the Chemogenomic Web Application https://ggshiny.shinyapps.io/2020NitrosoMechanisms/.

### Growth of strains deleted for amino acid metabolism genes is perturbed by N-nitrosamines

A striking finding in many of our screens was the effect of N-nitrosamines on strains lacking genes involved in many steps of amino acid biosynthesis in general, and arginine biosynthesis, in particular (Figs. 2, 3). For example, treatment with NDMA and NDEA caused reduced growth of *ARG1*, *ARG3*, *ARG5,6*, *ORT1*, *CPA1* and *CPA2* deletion strains. NDMA and NDEA also reduced the growth of a strain deleted for *GCN4*, which regulates expression of amino acid biosynthetic genes. Other arginine metabolism deletion strains that showed decreased growth upon exposure to NDMA exclusively were *ARG2*, *ARG4* and *ARG7*. In addition, NDMA caused growth defect of the *PRO1* mutant, this gene encodes gamma-glutamyl kinase, an enzyme of the proline biosynthetic pathway. NDEA caused fitness defects on mutants lacking genes of the tryptophan biosynthesis (*TRP1*, *TRP3*, *TRP4* and *TRP5*) and cysteine biosynthesis (*CYS3* and *CYS4*).

### Deletion strains of the arginine biosynthetic pathway genes are hypersensitive to DMF

**Figure 4:**
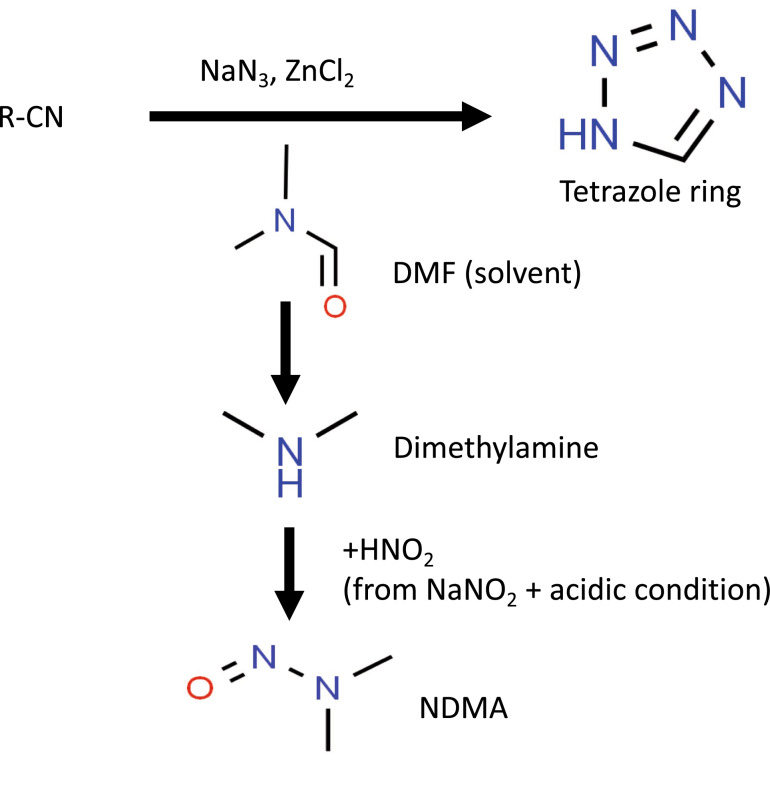
Proposed chemistry of NDMA formation from DMF during sartan production. The active pharmaceutical ingredient in sartan drugs has a tetrazole ring and the conventional method to produce this ring was reported to be slow. In order to accelerate the production process, a synthesis procedure that uses the solvent, dimethylformamide (DMF) and sodium azide (in place of tributyltin azide) was introduced in 2012. During tetrazole synthesis, a small amount of dimethylamine would be formed from DMF. The synthetic process also involves the use of nitrous acid to dispose of the excess sodium azide. This nitrous acid (a nitrosating agent) which is produced from sodium nitrite under acidic conditions can react with dimethylamine to form NDMA. (R = aryl, alkyl or vinyl; DMF = dimethylformamide; NDMA = N-nitrosodimethylamine). Figure adapted from^2,53–55^.

During the investigation into the source of N-nitrosamine contamination, several lines of evidence pointed to a manufacturing switch to the solvent DMF^2,3^ for the production of the APIs (Fig. 4) as a culprit. We therefore performed genome-wide screens on DMF as described for the three N-nitrosamines and found growth was impaired in strains deleted for the arginine metabolic genes *ARG1*, *ARG2*, *ARG3*, *ARG4*, *ARG5,6*, *ARG7*, *CPA1*, *CPA2* and *ORT1* (Fig. 2D). The *GCN4* deletion strain, as noted above, also had reduced fitness in DMF. Mutants deleted for genes involved in ATP export (e.g. *SNF7*, *SNF8*, *STP22*, *VPS28*, *VPS20*, *VPS25*, *DID4*) and vacuolar protein sorting (e.g. *SNF8, VPS25, VPS8, VPS27*) showed reduced growth in DMF. Other affected mutants include those lacking genes of the fatty acid biosynthesis (*CEM1*, *ELO3*, *HTD2*, *MCT1* and *PPT2*), protein lipoylation (*AIM22*, *GCV3*, *LIP2* and *LIP5*), and biotin biosynthesis pathways and iron–sulfur cluster assembly (*ISA1*, *ISA2* and *IBA57*). Overall, 58 deletion strains showed growth defects on exposure to DMF, and 27 of these strains showed reduced growth in NDMA and NDEA.

### Screening of metabolic intermediates of N-nitrosamines highlights the role of intracellular ammonia

**Figure 5:**
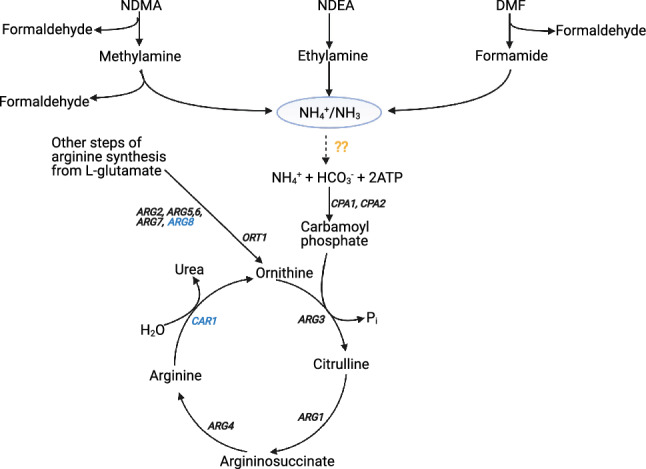
Relationship between nitrosamine/DMF degradation and arginine metabolism as observed in our experiments. Genes labelled in black correspond to deletion strains that displayed growth defects in our experiments, while those in blue did not show fitness defects. The dashed arrow with question marks (in yellow) means the reaction is hypothetical, i.e. depicting NH_4_ from nitrosamine degradation entering the arginine biosynthetic pathway. In the diagram, only metabolic intermediates of nitrosamine/DMF that we screened are shown.

**Figure 6:**
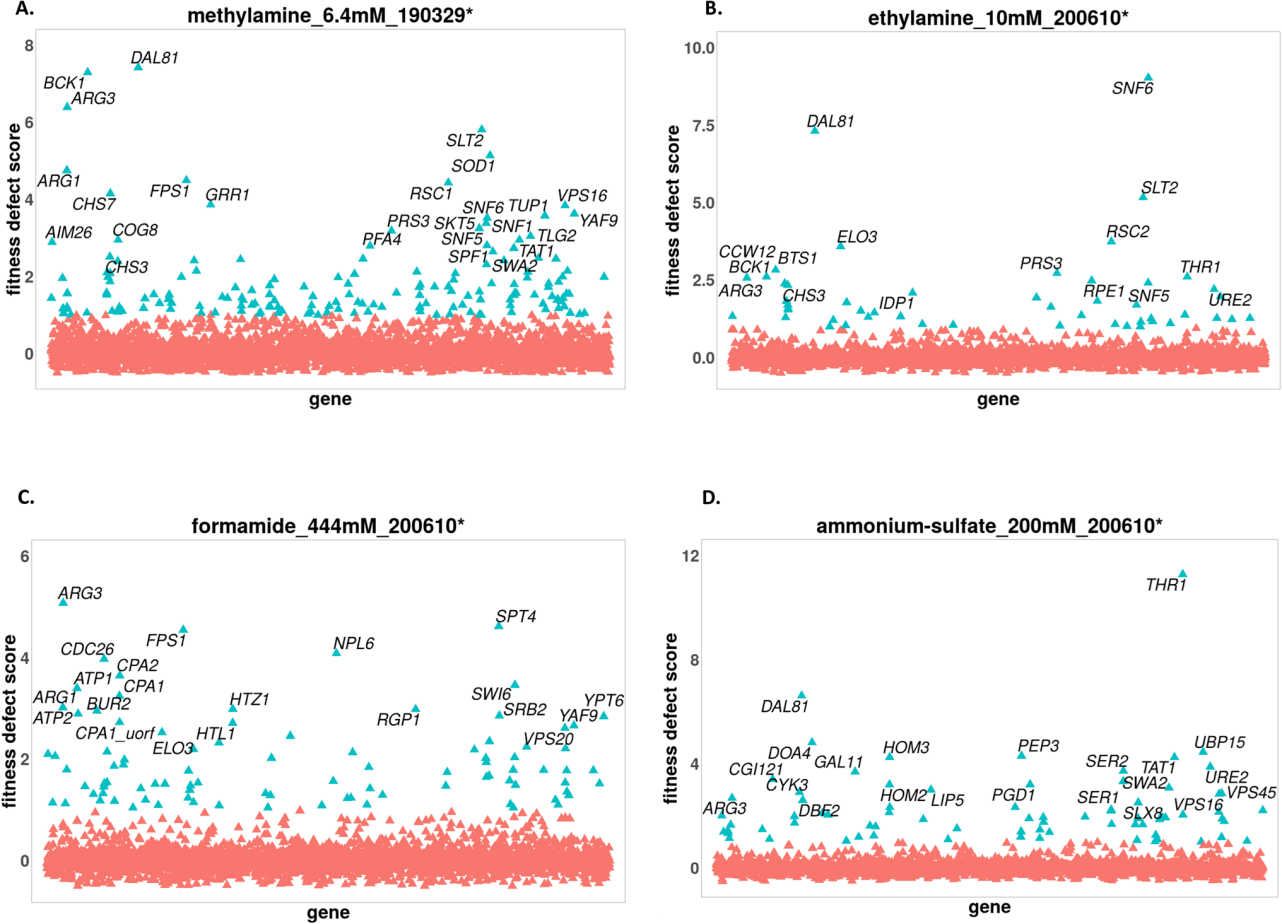
Fitness profile showing genes whose deletion strains are sensitive to treatment by metabolic intermediates of nitrosamines. Fitness defect scores are plotted on the vertical axis vs genes on the horizontal axis. Strains affected by the compounds lack the following genes; methylamine: *BCK1*, *DAL81*, *ARG3*, *FPS1* and *GRR1*. Ethylamine: *DAL81*, *ARG3*, *SNF6*, *ELO3*, *SNF6* and *ELO3*. Formamide: *ARG3*, *FPS1*, *NPL6* and *SPT4*. Ammonium sulfate: *DAL81*, *ARG3*, *PEP3*, *THR1*, etc. It could be seen that *ARG3* was affected by all the metabolites, in addition to *ARG1* being affected by methylamine and formamide. Concentration and date of count matrices analysis are indicated. Not all strains with significant FD scores are labelled. Plots were generated using the Bioconductor v3.12, https://www.bioconductor.org/.

Because several N-nitrosamines, as well as DMF, are known to generate ammonia through their metabolic intermediates in animals^17–19^, we suspected that additional ammonia produced during metabolism of N-nitrosamines might contribute to the toxic effects of NDMA, NDEA and DMF on deletion strains lacking genes of the arginine biosynthetic pathway (Fig. 5). We screened three intermediates: methylamine, ethylamine and formamide, as well as ammonium sulfate to test the relationship between ammonia production and excretion with arginine metabolism. Our data showed that *ARG3* deletion strain exhibited fitness defects in the presence of these compounds (Fig. 6). Both formamide and methylamine caused growth defects in *ARG1* and *ORT1* mutants, while methylamine caused defects in *ARG2*, *ARG4* and *ARG5,6* (Table 1). Similar to NDMA, NDEA and DMF, strains deleted for *CPA1* and *CPA2* also showed growth defects in formamide, but not in methylamine or ethylamine. Exogenous ammonium sulfate caused growth defects in strains deleted for *ARG3* but that for *CPA1* nor *CPA2* (Fig. 6).

**Figure 7:**
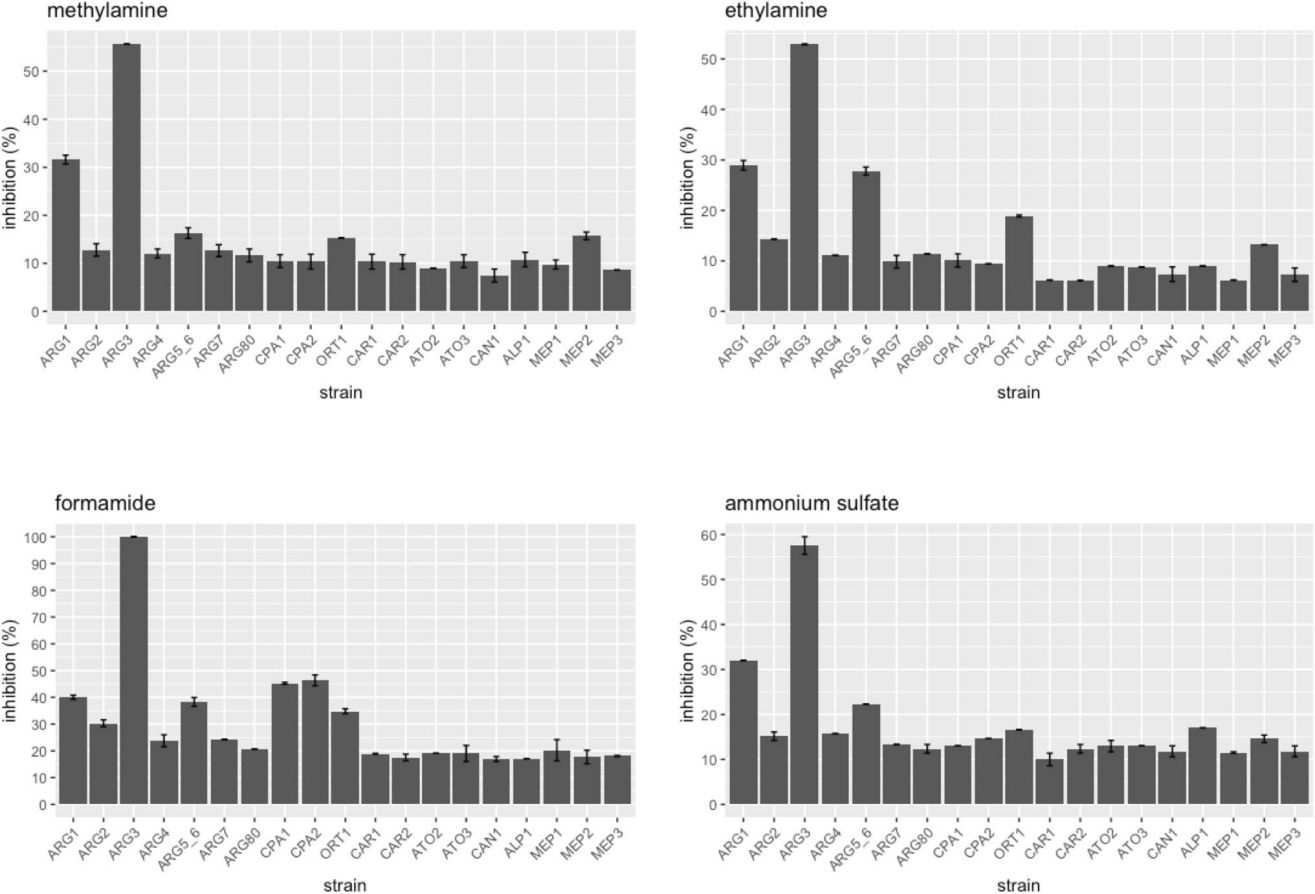
Percent inhibition of the intermediates against nineteen strains lacking arginine and ammonium metabolism genes. Strains lacking *CPA1* and *CPA2* were sensitive to formamide, but not to methylamine, ethylamine and ammonium sulfate. However, strains that are missing *ARG1*, *ARG3* and *ARG5,6* have increased sensitivity to the four compounds. Each bar represents the mean ± standard of inhibition (%).

**Figure 8:**
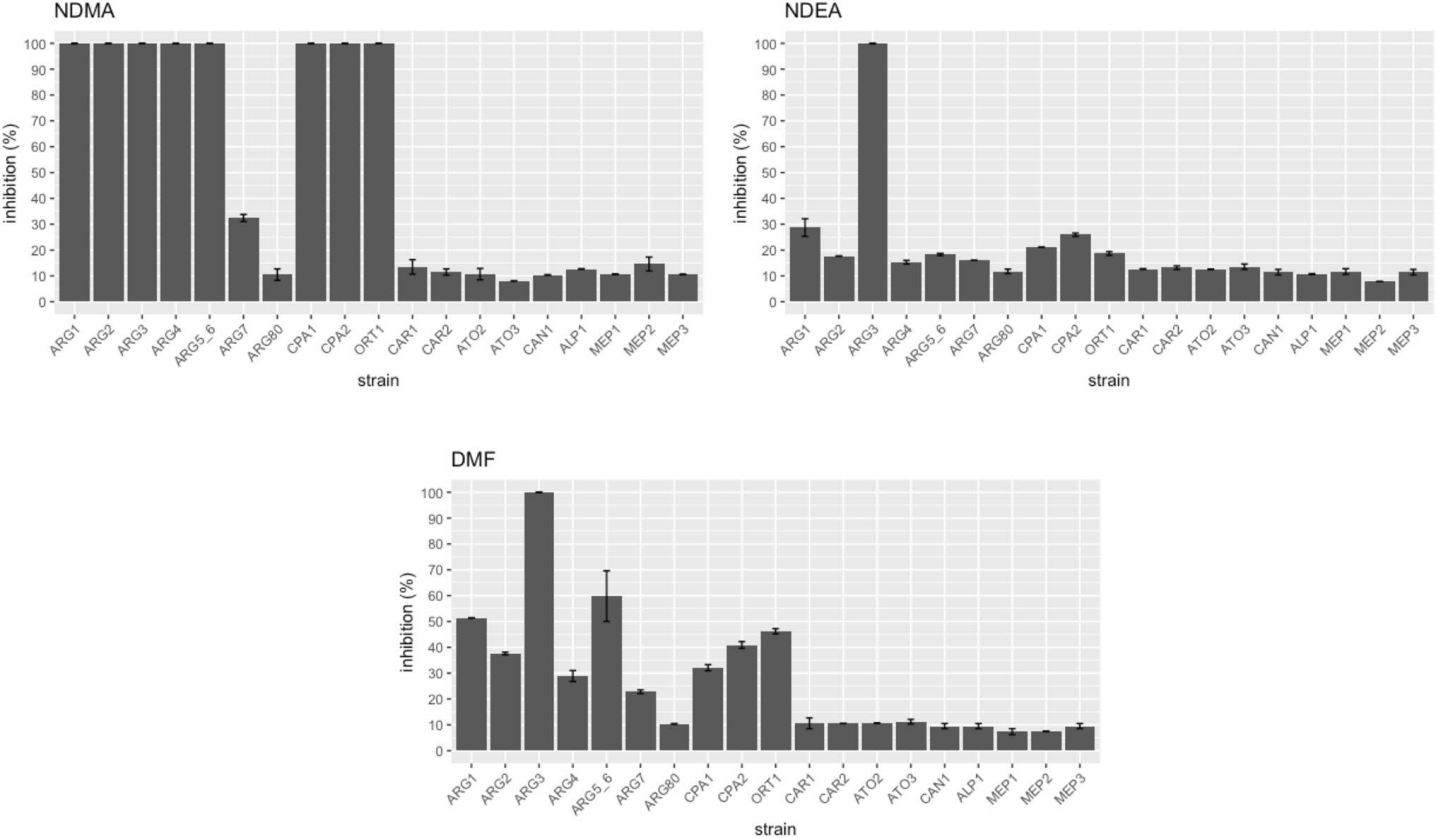
Percentage inhibition of the 19 arginine/ammonium deletion strains by N-nitrosamines and DMF. All the compounds showed greater  effects on *ARG1*, *ARG3*, *CPA1* and *CPA2* compared to other genes.

To confirm the results of our genome-wide screens, we selected nineteen individual deletion strains from our screens and tested them against all 10 compounds. The data, expressed as percent inhibition relative to DMSO or water control, confirmed the strain sensitivities observed from our pooled screening results. Similar to the genome-wide screen, the result showed methylamine, ethylamine and ammonium sulfate did not cause growth defects of strains deleted for *CPA1* and *CPA2* (Fig. 7). These individual strain tests also uncovered an effect on *ARG1* by ethylamine and ammonium sulfate which was not evident from the chemogenomic assay results. Additionally, the data confirmed our finding on the hypersensitive effect of NDMA, NDEA and DMF on arginine biosynthetic mutants (Fig. 8).

Given the large number of deletion strains in the arginine biosynthetic pathway that were affected by N-nitrosamine treatment, we tested if these mutants were unable to import arginine from the media. Accordingly, we co-administered NDMA with exogenous arginine (625 µM, 1.25 mM, 2.5 mM, 5 mM, 10 mM and 20 mM) on both the parental strain (BY4743) and an *ARG3* deletion mutant. Excess arginine did not relieve NDMA-induced inhibition of cell growth at any of the doses tested (Supplementary fig S1) suggesting that the growth defect is not simply due to a defect of arginine import.

### Genes affected by N-nitrosamine metabolic intermediates belong to diverse cellular pathways

**Figure 9:**
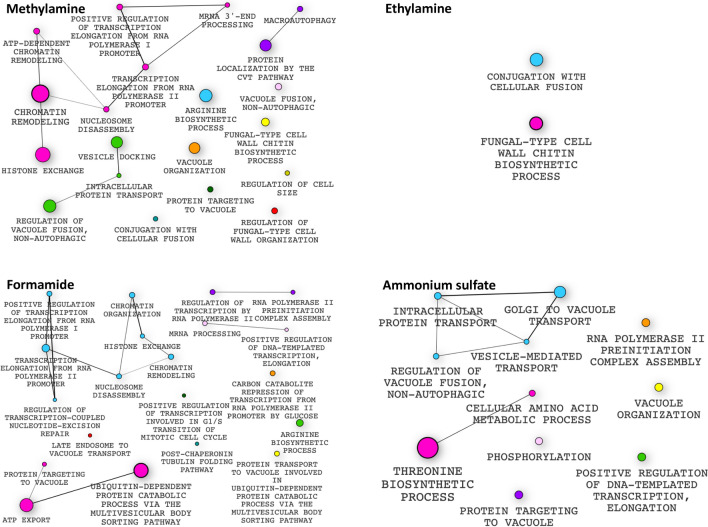
Gene ontology enrichment profiles showing pathways affected by the metabolic intermediates of N-nitrosamines. Genes deleted in strains that displayed increased sensitivity were enriched for certain biological processes including; methylamine include: chromatin remodeling, protein targeting to vacuole, microautophagy and arginine biosynthetic process. Ethylamine: cell wall chitin biosynthetic process and conjugation with cellular fusion. Formamide: arginine biosynthetic process, chromatin remodeling, ATP export and protein transport. Ammonium sulfate: protein targeting to vacuole, phosphorylation and cellular amino acid biosynthetic process. Enrichment profiles were generated using the Bioconductor v3.12, https://www.bioconductor.org/.

Overall, we found that 186 deletion strains displayed fitness defects upon exposure to methylamine. Formamide induced growth defects in 102 strains, while ethylamine and ammonium sulfate caused growth defects on 60 and 84 strains respectively. When their GO profiles were examined (Fig. 9), we found that methylamine caused growth defects in mutants in chromatin remodeling (*YAF9*, *SNF6*, *SNF5*, *SNF2*, *SWC5*) and protein transport (*CHS7*, *VPS16*, *TLG2*, *COG8*, *PEP3*), while ethylamine decreased the growth of mutants deleted for genes involved in chromatin remodeling (*SNF5*, *SNF6*, *RSC2*), fatty acid biosynthesis (*ELO3*) and amino acid biosynthesis (*THR1*, *HOM2*, *PRS3*). Similarly, formamide caused fitness defects of strains lacking chromatin remodeling genes (e.g. *HTZ1*, *HTL1*, *YAF9*, *ARP6*, *SWR1*), ATP export (e.g. *VPS20*, *VPS28*, *SNF7*, *SNF8*, *STP22*), vacuolar protein sorting (e.g. *VPS28*, *VPS25*, *STP22*, *SNF8*) and amino acid biosynthesis (*ARO2*, *CYS4*), while ammonium sulfate caused growth defects in mutants involved in amino acid biosynthesis (*THR1*, *HOM2*, *HOM3*, *HOM6*, *THR4 SER1*, *SER2*), vacuolar protein sorting (e.g. *VPS45*, *VPS16*, *VPS1*, *APL6*) and protein folding (*ZUO1*, *ALF1*, *SSE1*, *GSF2*, *FMO1*, *YME1* (Table 1; Figs. 6, 9). Methylamine and formamide caused growth defects in *GCN4* deletion mutants*.* Deletion of *DAL81* a transcription factor involved in nitrogen metabolism in yeast caused a growth defect in methylamine, ethylamine and ammonium sulfate. Growth defects are also caused by deletion of *URE2* which encodes a protein involved in nitrogen catabolite repression^20^ and which downregulates the expression of genes involved in nitrogen utilization by inhibiting Gln3p and Gat1p^21^.

Both ascorbic acid and ferulic acid have been proposed as potential remediators of N-nitrosamine toxicity—owing to the fact that these compounds are potent inhibitors of endogenous nitrosation^22–24^. We therefore co-administered NDMA or NDEA with each acid in our genome-wide screens. Our results showed that neither ascorbic acid nor ferulic acid (either individually or in combination) reduced the effect of NDMA and NDEA based on the number of sensitive strains detected. Control screens with ascorbic acid or ferulic acid in the absence of N-nitrosamines did not induce sensitivity on their own.

### Strains deleted for genes involved in DNA repair are sensitive to formaldehyde and 4NQO

**Figure 10:**
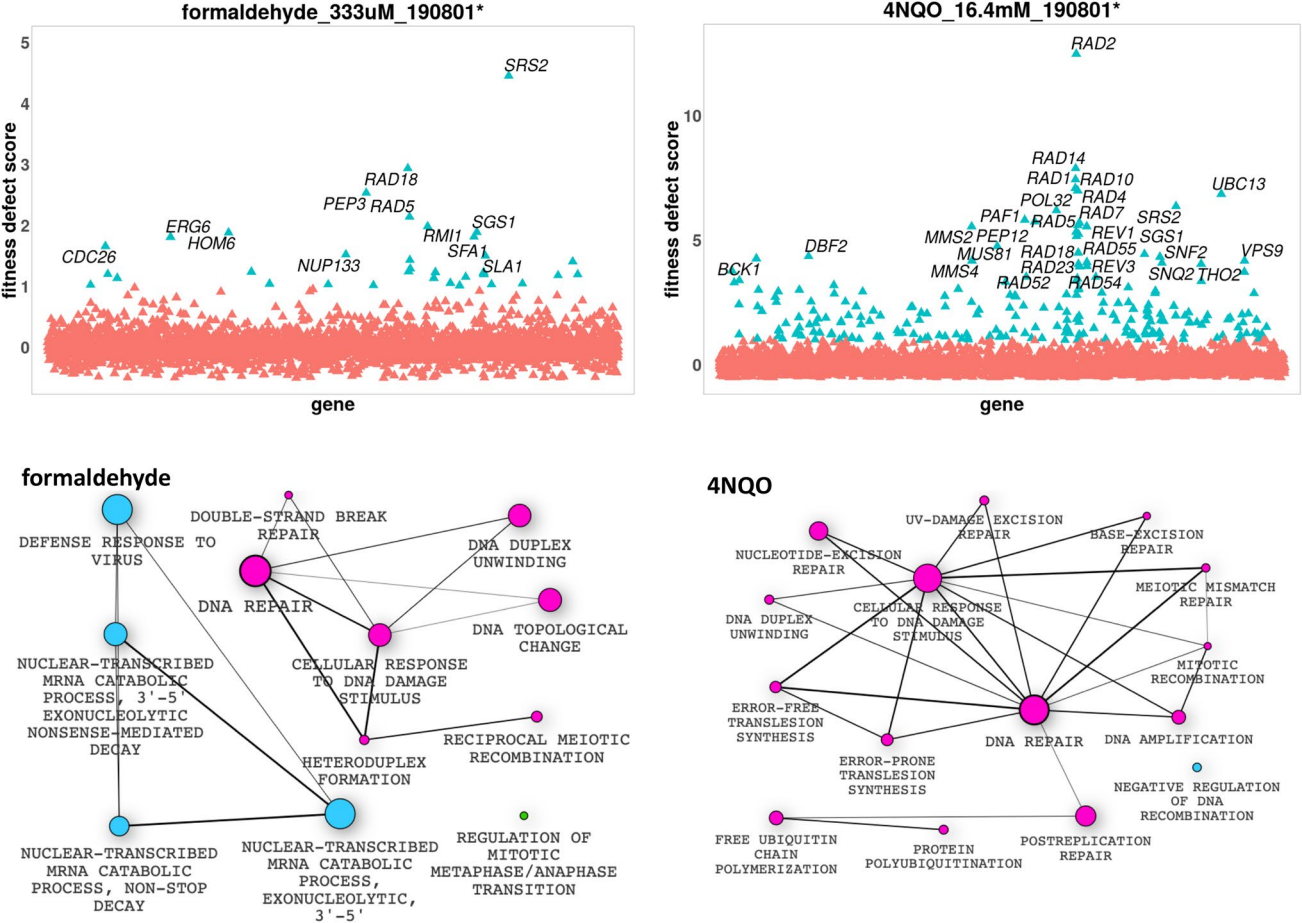
Genes and pathways whose deletion strains were affected by formaldehyde and 4NQO. Formaldehyde sensitized strains lacking *CDC26*, *RAD5* and *RAD18* which are involved in DNA repair process. Similarly, 4NQO induced sensitivity for strains deleted for *RAD2*, *RAD14*, *RAD10*, *MMS2* and *MUS81*, involved in DNA repair. As can be seen from the figure, many affected genes are those involved in cell cycle and DNA repair. Not all pathways at significant FD score are shown, e.g. chromatin remodelling in 4NQO. Plots were generated using the Bioconductor program v3.12, https://www.bioconductor.org/.

Because formaldehyde is one of the metabolic intermediates of NDMA, DMF and methylamine^25,26^ (Fig. 5), we tested formaldehyde in a chemogenomic screen and found that formaldehyde caused growth defects in 36 strains, many of which are deleted for genes involved in DNA repair (*SRS2*, *MUS81*, *RRD1*, *RAD5*, *RAD18*, *RAD27*, *RAD51*, *RAD55*, and *SGS1*) (Fig. 10). This observation may be explained by the known activity of formaldehyde in cross-linking proteins to DNA^27^. As expected, the *SFA1* deletion strain had growth defects in formaldehyde. *SFA1* encodes formaldehyde dehydrogenase which is known to be involved in detoxifying formaldehyde in yeast^28^.

Treatment with 4NQO, a known genotoxin, caused growth defects in 252 strains, including those lacking genes of the DNA repair pathways such as *RAD1*, *RAD2*, *RAD4*, *RAD5*, *RAD7*, *RAD10*, *RAD14*, *RAD18* and *SRS2*. Similarly, strains deleted for cell division genes, such as *BIM1*, *PAT1*, *RMI1*, *CDC10*, *ELM1* and *MEC3* displayed fitness defects upon 4NQO treatment. Furthermore, mutants deleted for genes of the chromatin remodeling pathway showed growth defects in 4NQO, which include *ARP5*, *ARP6*, *ARP8*, *FKH2*, *IES6*, *MRN1*, *RAD54*, *SNF2*, *SNF5* and *UME6*. Finally, strains deleted for ATP export genes (*SNF8, STP22, VPS28, SNF7* etc.) and vacuolar protein sorting genes (*VPS9, SNF8*, *STP22*, *VPS8*, *VPS25*, *VPS28*) also exhibited fitness defects in 4NQO (Fig. 10). Similar to formaldehyde, many of the strains deleted for several DNA repair and cell cycle genes showed growth defects in 4NQO.

### Comparisons across all compounds: cofitness and coinhibition provide insights into N-nitrosamine toxicity

**Figure 11:**
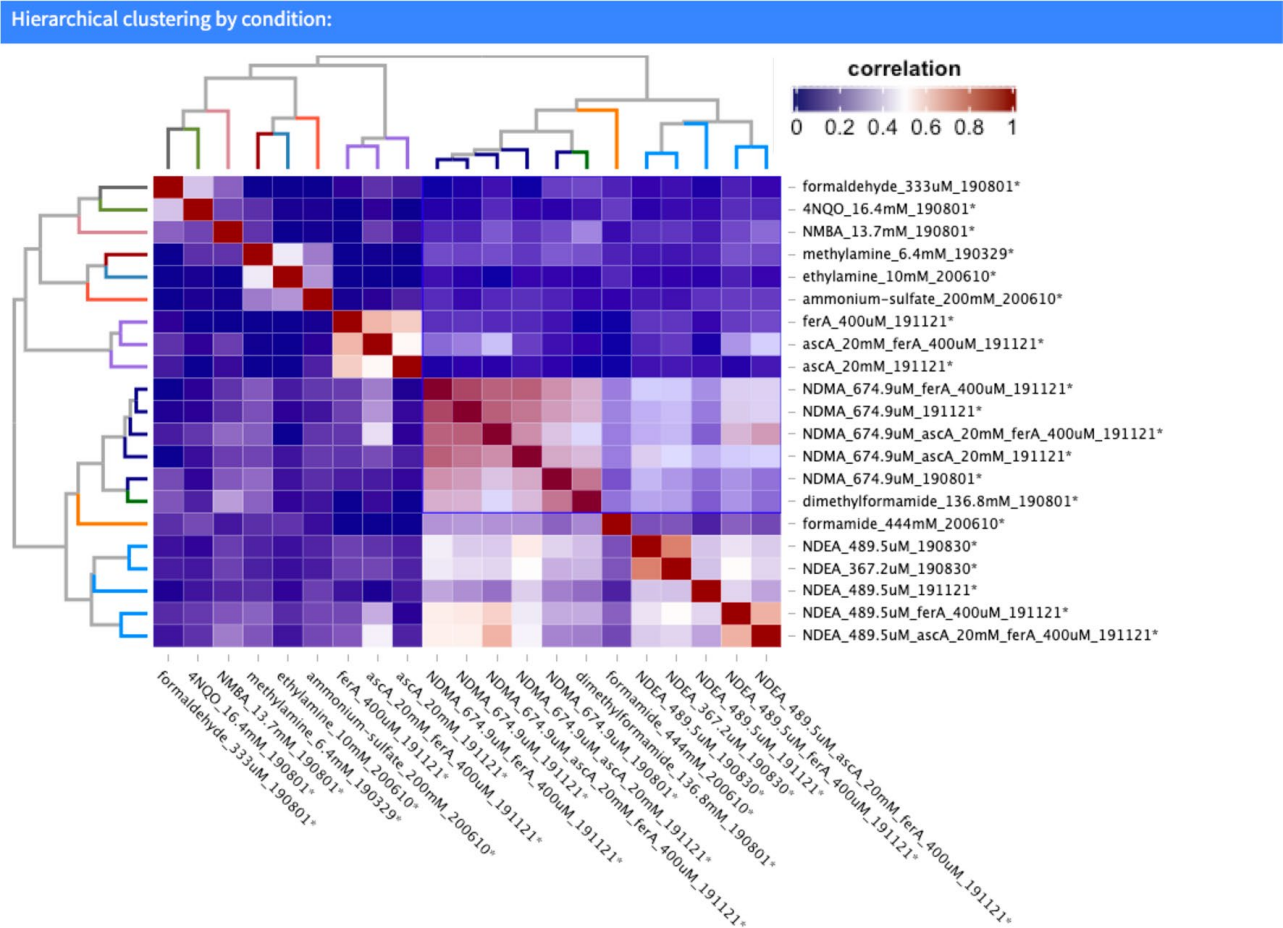
Hierarchical cluster analysis of the all 22 chemogenomic screens. Pearson correlation was used to generate the “coinhibitory” square matrix and Ward was used as the distance. Branch colors on the two identical dendrogram indicate the three major clusters. Compounds within each cluster are highly correlated as indicated by the color scale as well as the dendrogram height and may correspond to compounds that have similar chemogenomic profiles and therefore may suggest similar mechanism of action. For example, the fitness profiles for NDEA all cluster tighter as expected (orange dendrogram branch color). Globally, two of the three major clusters (orange and navy branches in dendrogram) that include NDMA, NDEA, DMF and formamide exhibit significant correlation (visualized as darker red region in the heatmap). This finding is consistent with the chemogenomic profile similarity between these compounds, all of which were enriched for the genes involved in the arginine biosynthetic pathway. Figure was generated using the Bioconductor v3.12, https://www.bioconductor.org/.

Different metrics can be used to determine similarities and differences between a large number of genome-wide profiles. For example, variability is used to calculate distance as represented by the correlation between samples. One commonly used measure of correlation is the Pearson coefficient which reflects the linear relationship between any two variables, accounting for differences in their mean and standard deviation (SD)^29^. The more similar the profiles for all fitness defects are between two samples, the higher the correlation coefficient^13^. We calculated these correlation coefficients for all samples and visualized the results as a heat map. These results are presented in Fig. 11 and in the supporting web application. This visualization highlights the similarities between 1) NDMA, NDEA, DMF and formamide and between 2) formaldehyde and 4NQO. Furthermore, it is noteworthy that each NDMA and NDEA replicate clusters adjacent to each other. Extending the global comparison between screens, we quantified this comparison (aka “cofitness”) based on their Pearson correlation as described^30^. Genes that exhibit a high degree of cofitness between fitness profiles are often functionally related^13,30^. The arginine biosynthetic genes, by way of example, show high levels of co-fitness. Similarly, coinhibition between profiles is defined by the Pearson correlation between chemical profiles, which can identify compounds that act by similar mechanisms, such as 4NQO and formaldehyde. The co-fitness of all the 4800 genes and coinhibition profiles of the 10 compounds in our experiment are accessible on the web application.

## Discussion

We present a comprehensive and unbiased portrait of genes and pathways, that when deleted, show growth defects when treated with compounds that have been implicated in the recent global recall of millions of doses of contaminated medications. By systematically screening these compounds, we provide insights into their effects on cells and possible mechanisms of toxicity. Furthermore, by presenting these results along with an interactive web application https://ggshiny.shinyapps.io/2020NitrosoMechanisms/, we equip researchers with the tools needed to perform their own analyses of global fitness defects, Gene Ontology (GO) enrichment and cofitness and coinhibition. The GO enrichment data which summarizes the affected pathways for all the compounds, is additionally provided in Supplementary file S1.

We observed that NDMA, NDEA and DMF caused growth defects in strains lacking arginine biosynthetic genes. These compounds all share the ability to produce ammonia through their metabolic intermediates. For example, denitrosation of NDMA is reported to produce methylamine which is further metabolized to ammonia and formaldehyde^17,26^. Similarly, denitrosation of NDEA produces nitrites and ethylamine^18,31^, and ethylamine is enzymatically transformed into ammonia and acetaldehyde^32,33^. DMF is metabolized to formamide, which would also likely increase the concentrations of intracellular ammonia and formic acid^19,22^. Given the multiple paths to produce ammonia in vivo we therefore tested these metabolic intermediates (as well as ammonium sulfate as a proxy for ammonia) to ask if ammonia played a role in the effect we observed on the strains lacking arginine biosynthetic genes. While methylamine and formamide decreased the growth of strains lacking arginine biosynthetic genes similar to their parent compounds, ethylamine and ammonium sulfate only caused growth defects in the *ARG3* deletions strain, whose wild type gene codes for the ornithine carbamoyltransferase enzyme that catalyzes the formation of citrulline from ornithine and carbamoyl phosphate. Carbamoyl phosphate itself is synthesized from ammonium and bicarbonate in an ATP-dependent reaction catalyzed by carbamoyl phosphate synthetase (*CPA1* and *CPA2*). Four of the compounds (NDMA, NDEA, DMF and formamide) caused growth defects in strains deleted for *CPA1* and *CPA2*, but methylamine, ethylamine and ammonium sulfate did not. The latter was unexpected, given the known roles of *CPA1* and *CPA2* in ammonia metabolism. Consistent with our observations, a published gene expression analysis reported that several amino acid genes, including those involved in arginine biosynthesis, were highly expressed in yeast in response to increased ammonia in the growth medium^34^. The fact that many amino acid biosynthetic mutants displayed growth defects in the presence of all compounds (except formaldehyde) (Table 1) is consistent with a disruption of amino acid biosynthesis or uptake. Although the *GCN4* mutant did not show growth defects in ammonium sulfate, ethylamine, NMBA and 4NQO, these compounds did affect growth of several amino acid biosynthetic mutants that are regulated by *GCN4*^35^.

The effects of NDMA and NDEA on mitochondrial mutants, specifically those involved in mitochondrial translation and genome maintenance, appear to be independent of their metabolic intermediates (i.e. methylamine and ethylamine respectively) because neither of these metabolites caused growth defects in mitochondrial deletion mutants. One potential explanation for this observation is that there are two biotransformation pathways for N-nitrosodialkylamines; α-hydroxylation (activation) and denitrosation. Methylamine and ethylamine are produced via the denitrosation route, whereas methyldiazonium and ethyldiazonium ions are formed in the activation pathway^36,37^. These diazonium ions are known to form DNA adducts via alkylation^9,36,37^. Our findings are consistent with an additional action of N-nitrosamines on mitochondrial function^38^. In addition to their essential roles in ATP production, mitochondria are also involved in heme production, iron–sulfur cluster assembly and calcium regulation^39^. In our screens, both NDMA and DMF affected mutants involved in iron–sulfur assembly as well as those of lipoic acid synthesis. Because lipoic acid is an obligate cofactor of pyruvate dehydrogenase and α-ketoglutarate dehydrogenase^40,41^, downstream effects of N-nitrosamines on energy metabolism are not unexpected, and NDEA has previously been reported to impair ATP production and mitochondrial function in rat^38^.

In conclusion, we tested ten compounds in genome-wide experiments and characterized the differential growth of all homozygous deletion mutants grown in pooled culture. Given the ubiquity of these compounds in nature and in diverse industrial processes including pharmaceutical production, a better understanding of the cellular effects of these toxins is crucial. Among the conserved pathways uncovered in this study, we were able to determine that deletion of genes involved in arginine biosynthesis renders mutants sensitive to the effects of NDMA, NDEA and DMF. It is important to note that, despite the conservation of many of the pathways identified in this study, the results obtained with the yeast model system may not be directly transferable to metazoans—owing to complexities such as tissue specific metabolism. Nevertheless, this comprehensive dataset of deletion strains that are affected by exposure to N-nitrosamines and related compounds, combined with an accessible, queryable database represents a valuable resource that can provide new insights and inspire further work into the biological consequences of exposure to these contaminants.

## Methods

### Chemicals

N-nitrosodiethylamine (CAS: 55-18-5), N-nitrosodimethylamine (CAS 62-75-9) and N-Nitroso-N-methyl-4-aminobutyric acid (CAS 61445-55-4) were purchased from Toronto Research Chemicals; formaldehyde (CAS 50-00-0), N,N-dimethylformamide (CAS 68-12-2), formamide (CAS: 75-12-7) and ammonium sulfate (7783-20-2) were from Fisher Scientific; 4-Nitroquinoline-N-oxide (CAS 56-57-5), methylamine (CAS 74-89-5), ethylamine (CAS: 75-04-7), L-ascorbic acid (CAS 50-81-7) and trans-ferulic acid (CAS 537-98-4) were purchased from Sigma-Aldrich. All chemical compounds were stored as instructed by the manufacturer prior to use.

### Yeast deletion collection

The yeast (BY4743) diploid homozygous deletion collection, comprising 4800 non-essential deletion strains was commercially obtained from TransOmic Technologies Inc. Strains were robotically pinned onto YPD (10 g/L yeast extract, 20 g/L peptone and 20 g/L dextrose) + 200 mg/L G418 plates. Following 48 h of growth, the colonies were aseptically scraped with synthetic complete (SC) media^42^ (i.e. 20 g/L dextrose; 1.7 g/L yeast nitrogen base without amino acid and ammonium sulfate; 2.0 g/L amino acid and supplement mix (SC Complete Powder from Sunrise Science Products, Catalog # 1300-030); and 5.0 g/L ammonium sulfate). These colonies were used to make the strain ‘pool’, so that the pool contains approximately equal number of cells per strain. This pool was diluted to an optical density (OD_600_) of 50, aliquoted and stored at − 80 °C prior to use.

### Dose determination for chemogenomic screens

BY4743 wild type cells (the parent of the homozygous deletion collection) were incubated in SC media at 30 °C with continuous shaking. After 20 h of growth, cells were diluted to an OD_600_ 0.0625. A seven-fold 1:2 dilution of the stock concentration of the compounds were made in DMSO (NDEA, NDMA, NMBA, DMF and 4QNO) or water (methylamine, ethylamine, formamide, formaldehyde and ammonium sulfate). Next, 2 µL of each dilution was added into respective wells of 96-well microtiter plate containing 98 µL of the BY4743 cell suspension, with DMSO or water serving as control. While we and others routinely use DMSO as a drug diluent^30,43,44^ it is worth noting that, although low concentrations of DMSO (< 2%) do not affect cell growth^45^, there are effects on gene expression^46^. Regardless, in these assays all growth was normalized to the DMSO reference. The plate was sealed with transparent tape and incubated using the Tecan GENios microplate reader (Tecan U.S) at 30 °C with continuous shaking for 20 h. OD_600_ readings were taken every 15 min and each sample was assayed in a duplicate. Data were collected and percentage inhibition estimated using the AUDIT software^47^. After calculating the concentrations that produced 10–20% inhibition of the wild type, we used the following final concentrations for the global screening of the deletion strains: NDMA 674.9 µM, NDEA 489.5 µM, NMBA 13.7 mM, DMF 136.8 mM, methylamine 6.4 mM, ethylamine 10 mM, formamide 444 mM, ammonium sulfate 200 mM, formaldehyde 333 µM and 4NQO 16.4 mM. Due to solubility issues, we used 20 mM ascorbic acid and 400 µM ferulic acid.

### Chemogenomic assays

The pool of deletion strains was diluted with SC media to an OD_600_ 0.0625 and transferred into the wells of 48-well microtiter plates. Each test compound was added at final concentrations that showed 10–20% growth inhibition against the wild type (in a total sample volume of 700 µL. The control DMSO or water was added at final 2% v/v. Cells were incubated using the Tecan GENios microplate reader (Tecan U.S) at 30 °C with continuous shaking. Following incubation for 20 h, the samples were collected, cells pelleted in microcentrifuge tubes and stored at − 20 °C prior to genomic DNA isolation. Each sample experiment was done in a biological triplicate.

### Toxicity rescue assay/antidote screening

NDMA or NDEA (at 10–20% growth inhibition to the wild type) was added to homozygous deletion pools diluted at an OD_600_0.0625 in wells of 48-well microtiter plate on SC media. This was followed by the addition of ascorbic acid and/or ferulic acid at a final concentration of 20 mM and 400 µM respectively, in a final sample volume of 700 µL. Following 20 h incubation at 30 °C with continuous shaking, the samples were removed and kept at − 20 °C prior to genomic DNA extraction.

For extracellular arginine experiments, BY4743 and the *ARG3* deletion mutant were independently incubated with 674.9 µM NDMA (a concentration that inhibited BY4743 by 15%) and/or arginine (at different final dose of 625 µM, 1.25 mM, 2.5 mM, 5 mM, 10 mM and 20 mM). Each compound was added at 2% v/v in 700 µL total cell suspension using synthetic arginine dropout media^42^ (i.e. 20 g/L dextrose; 1.7 g/L yeast nitrogen base without amino acid and ammonium sulfate; 2.0 g/L amino acid and supplement mix (SC-Arg Powder from Sunrise Science Products, Catalog # 1346-030); and 5.0 g/L ammonium sulfate). The cells were incubated as described above.

### Genomic DNA extraction and PCR

Genomic DNA was extracted using the YeaStar genomic DNA kit D2002 (Zymo Research) according to the manufacturer's manual protocol I. Two independent 20 µL PCR reactions, one for the uptag and the other for the downtag, were set up with the following cycling conditions: 98 °C for 3 min, 25 cycles of 98 °C for 10 s, 59 °C for 30 s and 72 °C for 15 s, and final 72 °C for 5 min with 4 °C hold.

The uptag primers were 5′-TCGTCGGCAGCGTCAGATGTGTATAAGAGACAGGATGTCCACGAGGTCTCT-3′ (F) and 5′-GTCTCGTGGGCTCGGAGATGTGTATAAGAGACAGGTCGACCTGCAGCGTACG-3′ (R), while the downtag primers were 5′-GTCTCGTGGGCTCGGAGATGTGTATAAGAGACAGCGGTGTCGGTCTCGTAG-3′ (F) and 5′-TCGTCGGCAGCGTCAGATGTGTATAAGAGACAGGAAAACGAGCTCGAATTCATCG-3′ (R).

### Barcode sequencing and analysis

Barcode sequencing was performed as described in Acton *et al*^48^. Pooled sequencing libraries were sequenced on the NextSeq High Output flow cell (Illumina), generating single-end 50 bp reads. The sequencing was performed at the Sequencing and Bioinformatics Consortium, University of British Columbia, Vancouver, Canada.

### Fitness defect (FD) score

The barseq count matrix initially comprised 116 chemogenomic screens performed in triplicate for each compound at different concentrations performed on six different dates. To calculate the relative abundance of the barcodes or FD scores for each strain, UPTAGs and DOWNTAGs representing each ORF/gene in the final barcode count matrix were first summed and experiments selected for reproducibility. After quality control and removing screens performed at suboptimal dosing, the final count matrix included 104 screens. These replicates were collapsed, resulting in 22 independent screens shown on the accompanying website https://ggshiny.shinyapps.io/2020NitrosoMechanisms/. To calculate these FD Scores for these screens, or − log_2_ ratios, we used the DESeq function with default parameters followed by the lfcShrink function using the apeglm method (lfcThreshold = 1), from the R package DESeq2^49^.

### Gene ontology (GO) enrichment analysis

GO enrichment analysis was performed as described in Lee *et al*^13^. GO annotations were retrieved from the Saccharomyces Genome Database (downloaded November 27, 2020). Gene sets correspond to the Gene ontology (GO) defined biological processes. GO biological processes that were too specific (contain less than five genes) or too general (contain greater than 300 genes) were excluded from the analysis. For a given query set of genes (e.g., genes with significant FD scores in the screening profile), the hypergeometric test was used to obtain a P value estimating the significance with which the set is enriched with genes annotated to a given GO category. FDR values were obtained from the resulting P values using the Benjamini and Hochberg method of adjusting for multiple comparisons^50^.

For a given set of query genes, the number of GO terms significantly enriched (FDR < 0.1) in the set can result in many categories that are closely related. To facilitate interpretation for a given query, we pruned the set of enriched categories using an adaptation of the GO-Elite method^51^. Briefly, for categories in the same lineage in the GO hierarchy, we identified the most significantly enriched term and pruned the others. In addition, we assessed the genes that drove the enrichment of each remaining category, i.e., genes in the overlap of the query set and the category set. We clustered the remaining categories based on similarities in their overlap gene sets, measured by the Jaccard coefficient. For each cluster, we identified the most significantly enriched category and pruned the others. The GO enrichment results were visualized with enrichment maps (e.g., Fig. 3; all enrichment maps are available on the accompanying website https://ggshiny.shinyapps.io/2020NitrosoMechanisms/), generated in R (see https://github.com/ucheogbede/2020NitrosoMechanisms.git for R code). Overlap coefficients less than 0.5 were set to zero. Nodes in the same cluster were assigned the same node color. Moreover, the size of a node was made to be proportional to the significance with which the corresponding GO category is enriched [− log_10_(FDR)].

### Hierarchical clustering

Our chemogenomic dataset is in a matrix format where each screen is a column, and each row is a gene name. To identify robust clusters, we first compute coinhibition, the pairwise Pearson correlation between all screens, representing the similarity between profiled compounds. Profiles were then hierarchically clustered using hclust in R (1 − coinhibition as the distance metric) and the Ward agglomeration method^52^.

### Chemogenomic analysis web application

A file containing the codes needed for local installation of the web application is provided and has been deposited in GitHub https://github.com/ucheogbede/2020NitrosoMechanisms.git.

### Individual strain confirmation

Nineteen deletion strains of the arginine and ammonium metabolism (*ARG1*, *ARG2*, *ARG3*, *ARG4*, *ARG5,6*, *ARG7*, *ARG80*, *CPA1*, *CPA2*, *ORT1*, *CAR1*, *CAR2*, *ATO2*, *ATO3*, *CAN1*, *ALP1*, *MEP1*, *MEP2* and *MEP3*) were individually cultured and diluted to an OD_600_ of 0.0625 in SC media. They were individually grown with each of the compounds at the concentration as described above in 96MTP at a final sample volume of 100 µL. The control (DMSO or water) vs treatment (test compound) for each strain were performed in biological duplicate. The cell growth was done at 30 °C and OD robotically measured every 15 min for 20 h. AUDIT software^47^ was used to estimate percentage inhibition among strains for each compound. The ggplot2 in Rstudio was used for further statistical analysis of the data.

## Supplementary Information

Supplementary Information 1.

Supplementary Video 1.

Supplementary Information 2.

Supplementary Information 3.

## Data Availability

The count matrices and the OD versus time readings for the nineteen strain confirmation screens have been deposited in figshare https://doi.org/10.6084/m9.figshare.13250198. The fastq files are available from the corresponding author upon request.

## Abbreviations

NDMA: N-nitrosodimethylamine
NDEA: N-nitrosodiethylamine
NMBA: N-methyl-N-aminobutyric acid
DMF: N,N-dimethylformamide
4NQO: 4-Nitroquinoline-1-oxide
