# Supplementary material for: A genome-wide portrait of pervasive drug contaminants
Source: Sci Rep. 2021 Jun 14;11:12487. doi: 10.1038/s41598-021-91792-1 (PMC8203678; doi:10.1038/s41598-021-91792-1)
Supplement: Supplementary file 1 — Supplementary Information 1. [file 41598_2021_91792_MOESM1_ESM.docx]

Supplementary section

**Movie S1:** Video demonstrating the features of the web app for exploring the results of the present study.

**Figure S1: Addition of arginine to the media did not relieve NDMA toxicity.** (i) Wild type and (ii) *arg3* mutant cells were separately grown against NDMA in a media containing different doses of arginine. Inhibition (%) of cells by arginine only (i.e. no NDMA), NDMA only (no arginine) or NDMA plus arginine was estimated relative to control samples (i.e. cells grown on media with no addition of arginine and NDMA). It could be observed that arginine at 20 mM is 100% lethal to the yeast cells. NDMA concentration is the same in all samples, originally estimated at 15% inhibition to the BY4743 WT (although variations were observed when tested on *arg3* mutant as can be seen on the plots). The growth curve is plotted with optical density on the vertical axis versus time (hr). Values in each plot represent the percentage inhibition relative to control (with the control curve indicated in black and the different treatments indicated by color), with positive values indicating sensitivity (inhibition) while zero or negative numbers means no inhibition. arg = arginine; rep = replicate. For samples containing arginine (arg), A = 20 mM, B = 10 mM, C = 5 mM, D = 2.5 mM, E = 1.25 mM and F = 0.625 mM of arginine. Growth curves are generated using the AUDIT program^47^ v1.1 and accessible from <https://nicolascoutin.shinyapps.io/audit/>.

File S1: Gene Ontology enrichment data showing enriched pathways and for all the compounds in our screen
