# Supplementary material for: A genome-wide portrait of pervasive drug contaminants
Source: Sci Rep. 2021 Jun 14;11:12487. doi: 10.1038/s41598-021-91792-1 (PMC8203678; doi:10.1038/s41598-021-91792-1)
Supplement: Supplementary file 3 — Supplementary Information 2. [file 41598_2021_91792_MOESM3_ESM.pdf]

A genome-wide portrait of pervasive drug contaminants

Joseph Uche Ogbede, Guri Giaever and Corey Nislow

Supplementary figure 1

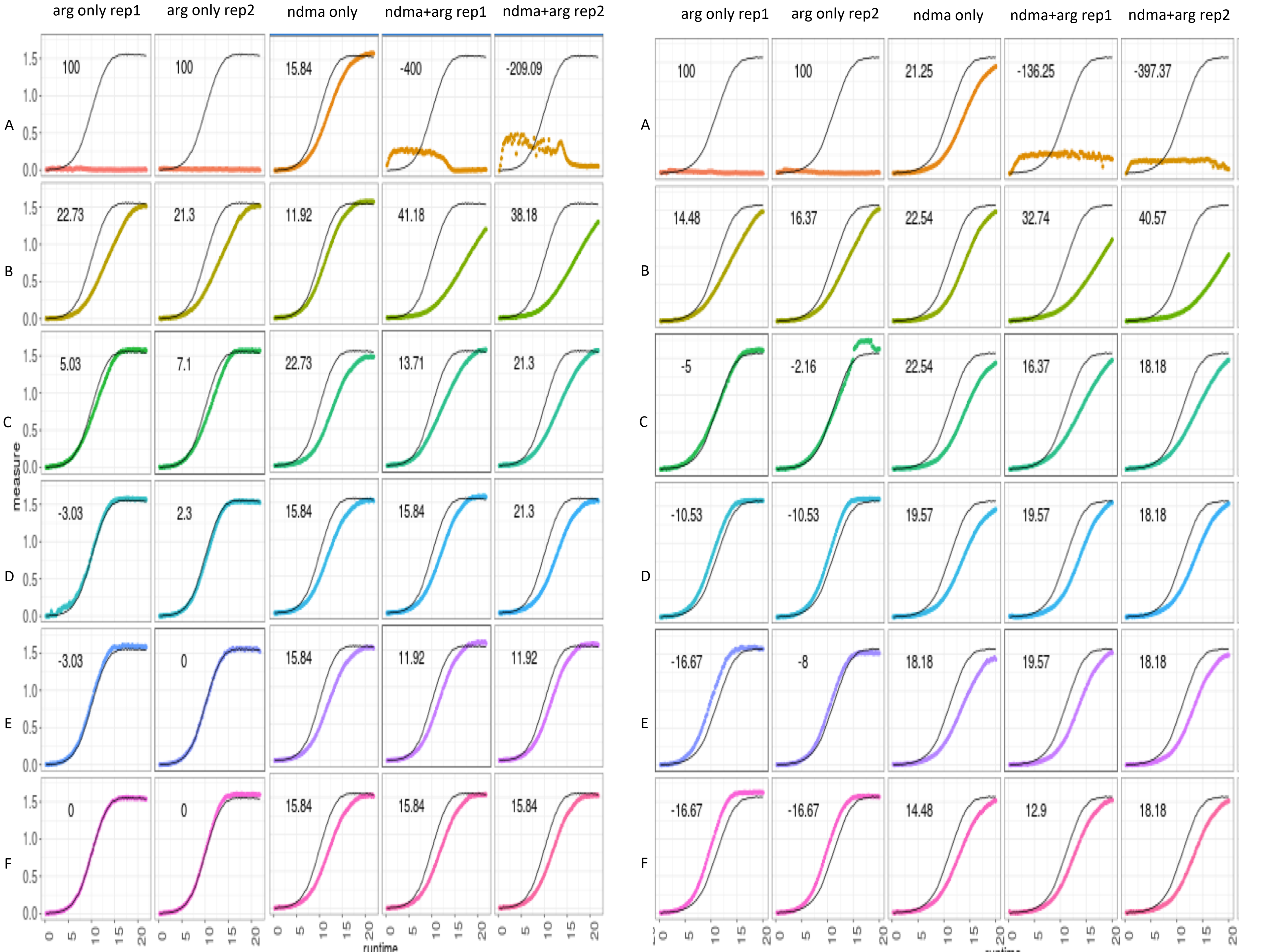

i. BY4743 WT

ii. *arg3* mutant
